# PRehabIlitatiOn with pReoperatIve exercise and educaTion for patients undergoing major abdominal cancer surgerY: protocol for a multicentre randomised controlled TRIAL (PRIORITY TRIAL)

**DOI:** 10.1186/s12885-022-09492-6

**Published:** 2022-04-22

**Authors:** Daniel Steffens, Jane Young, Bernhard Riedel, Rachael Morton, Linda Denehy, Alexander Heriot, Cherry Koh, Qiang Li, Adrian Bauman, Charbel Sandroussi, Hilmy Ismail, Mbathio Dieng, Nabila Ansari, Neil Pillinger, Sarah O’Shannassy, Sam McKeown, Derek Cunningham, Kym Sheehan, Gino Iori, Jenna Bartyn, Michael Solomon

**Affiliations:** 1 Surgical Outcomes Research Centre (SOuRCe), Royal Prince Alfred Hospital, PO Box M157, Missenden Road, Sydney, NSW 2050 Australia; 2 Faculty of Medicine and Health, Central Clinical School, The University of Sydney, Sydney, New South Wales Australia; 3 Faculty of Medicine and Health, Sydney School of Public Health, The University of Sydney, Sydney, New South Wales Australia; 4 Department of Anaesthesia, Perioperative and Pain Medicine, Peter MacCallum Cancer Centre and the Department of Critical Care, The Sir Peter MacCallum Department of Oncology, University of Melbourne, Melbourne, Victoria Australia; 5 Department of Health Economics & Health Technology Assessment, NHMRC Clinical Trials Centre, The University of Sydney, Sydney, New South Wales Australia; 6 Melbourne School of Health Sciences, University of Melbourne, Melbourne, Victoria Australia; 7 Department of Cancer Surgery, Peter MacCallum Cancer Centre, Melbourne, Victoria Australia; 8 Institute of Academic Surgery, Royal Prince Alfred Hospital, Sydney, New South Wales Australia; 9 Statistics Division, The George Institute for Global Health, University of New South Wales, Sydney, New South Wales Australia; 10 Department of Colorectal Surgery, Royal Prince Alfred Hospital, Sydney, New South Wales Australia; 11 Department of Anaesthetics, Royal Prince Alfred Hospital, Camperdown, New South Wales Australia; 12 Cancer Voices NSW, Sydney, New South Wales Australia

**Keywords:** Preoperative, Prehabilitation, Exercise, Education, Cancer, Surgery, Gastrointestinal, Complications, Randomised controlled trial

## Abstract

**Background:**

Radical surgery is the mainstream treatment for patients presenting with advanced primary or recurrent gastrointestinal cancers; however, the rate of postoperative complications is exceptionally high. The current evidence suggests that improving patients’ fitness during the preoperative period may enhance postoperative recovery. Thus, the primary aim of this study is to establish the effectiveness of prehabilitation with a progressive, individualised, preoperative exercise and education program compared to usual care alone in reducing the proportion of patients with postoperative in-hospital complications. The secondary aims are to investigate the effectiveness of the preoperative intervention on reducing the length of intensive care unit and hospital stay, improving quality of life and morbidity, and reducing costs.

**Methods:**

This is a multi-centre, assessor-blinded, pragmatic, comparative, randomised controlled trial. A total of 172 patients undergoing pelvic exenteration, cytoreductive surgery, oesophagectomy, hepatectomy, gastrectomy or pancreatectomy will be recruited. Participants will be randomly allocated to prehabilitation with a preoperative exercise and education program (intervention group), delivered over 4 to 8 weeks before surgery by community physiotherapists/exercise physiologists, or usual care alone (control group). The intervention will comprise 12 to 24 individualised, progressive exercise sessions (including aerobic/anaerobic, resistance, and respiratory exercises), recommendations of home exercises (16 to 32 sessions), and daily incidental physical activity advice. Outcome measures will be collected at baseline, the week prior to surgery, during the hospital stay, and on the day of discharge from hospital, and 1 month and 1 months postoperatively. The primary outcome will be the development of in-hospital complications. Secondary outcomes include the length of intensive care unit and hospital stay, quality of life, postoperative morbidity and costs.

**Discussion:**

The successful completion of this trial will provide robust and high-quality evidence on the efficacy of a preoperative community- and home-based exercise and education intervention on important postoperative outcomes of patients undergoing major gastrointestinal cancer surgery.

**Trial registration:**

This trial was registered prospectively with the Australian New Zealand Clinical Trials Registry (ACTRN12621000617864) on 24th May 2021.

## Background

For a selected group of patients presenting with advanced primary or recurrent cancers within the pelvis or abdomen, complex and extensive surgery may offer the only chance of long-term survival [1, 2]. Despite the survival benefit, the development of in-hospital postoperative complications after radical surgical resection with the intent of clear surgical margin remains very high [3]. For patients undergoing pelvic exenteration, cytoreductive and advanced upper gastrointestinal cancer surgeries, postoperative complication rates can range from 40 to 70% [4, 5] and comprise debilitating complications, including anastomotic leak, ileus, wound infection, sepsis, severe bleeding and pneumonia [6, 7]. These are major complications which require intensive treatment, prolonging hospital stay, reducing patient’s quality of life [1, 8, 9] and increasing the cost of care [10, 11].

Recent evidence suggests that the patient’s preoperative status largely determines the risk of developing postoperative complications following cancer surgery [12]. Patients presenting a low level of physical fitness during the preoperative period were more likely to develop postoperative complications [13, 14] while higher levels of self-reported physical activity were associated with improved postoperative outcomes [15]. Over the past decade, preoperative exercise has gained popularity, with the intent of strengthening functional capacity before surgery, accelerating the return to the preoperative conditions. To date, several randomised controlled trials (RCTs) have been conducted to investigate the effectiveness of a wide range of preoperative exercise programs in reducing postoperative complications and length of hospital stay in patients undergoing cancer surgery [16].

A recent systematic review and meta-analysis identified 13 unique clinical trials (including 806 patients) investigating the effectiveness of prehabilitation with preoperative exercise in patients that underwent surgical resection for colorectal, liver, oesophageal, lung, oral, and prostate cancer [17]. Most of the patient cohorts, comprised of single, small and underpowered trials. In patients undergoing lung cancer surgery, preoperative exercise effectively reduced postoperative complications by approximately 50% and hospital length of stay by almost 3 days compared to no treatment control. Other more recent trials reported similar results and strengthened this body of knowledge. For example, an RCT investigating the efficacy of prehabilitation, including endurance exercise and promotion of physical activity in a mixed group of 125 patients (including non-cancer patients) undergoing abdominal surgery, reported a significant increase in aerobic capacity and subsequent reduction in postoperative complications by over 50%, when compared to standard care [18]. Similarly, a multicentre, RCT involving 441 patients undergoing upper abdominal surgery investigated the effectiveness of preoperative breathing exercises [19]. This trial demonstrated a reduction of 52% in postoperative pulmonary complications in the preoperative intervention group compared to the control group.

Despite these previous studies providing evidence in support of preoperative exercise to improve postoperative outcomes, this evidence-base is limited by small sample sizes [20–24], the inclusion of mixed populations and heterogeneous surgical types (cancer/non-cancer) [18], comprehensive to state hospital-based intervention and exclusion of regional-rural patients who cannot travel [18] and other methodological flaws such as failure to conceal allocation and lack of intention-to-treat analyses. Therefore, the role of preoperative exercise for patients undergoing major advanced or recurrent gastrointestinal surgery is still unclear [25].

To overcome this paucity of robust evidence, our team conducted a pilot RCT, which demonstrated the feasibility and acceptability of a preoperative exercise program in patients undergoing major gastrointestinal cancer surgery to inform this large RCT: The Priority Trial [26].

The primary aim of this full-scale RCT is to establish the effectiveness of prehabilitation with a progressive, individualised, preoperative exercise and education program (compared to a usual care study arm) in reducing the proportion of patients with postoperative in-hospital complications within the primary admission of the index surgery. Our primary hypothesis is that the preoperative intervention with exercise and education will reduce the proportion of patients with postoperative in-hospital complications compared to usual care. The secondary aims are to investigate whether a preoperative exercise and education program, compared to usual care, reduces the length of intensive care and hospital length of stay, improves postoperative patient quality of life, and reduces postoperative morbidity. Furthermore, this trial seeks to determine the cost-effectiveness of the preoperative exercise and education program.

## Methods/design

This protocol was written in accordance with the Consolidated Standard of Reporting Trials (CONSORT) [27, 28], followed recommendations from the Standard Protocol Items: Recommendations for Interventional Trials (SPIRIT) Checklist [29, 30] and has been prospectively registered with the Australian New Zealand Clinical Trials Registry (ACTRN12621000617864). Ethics approval has been obtained from the Sydney Local Health District Human Research Ethics Committee – Concord Hospital (2019/ETH13718).

### Trial design

**Fig. 1:**
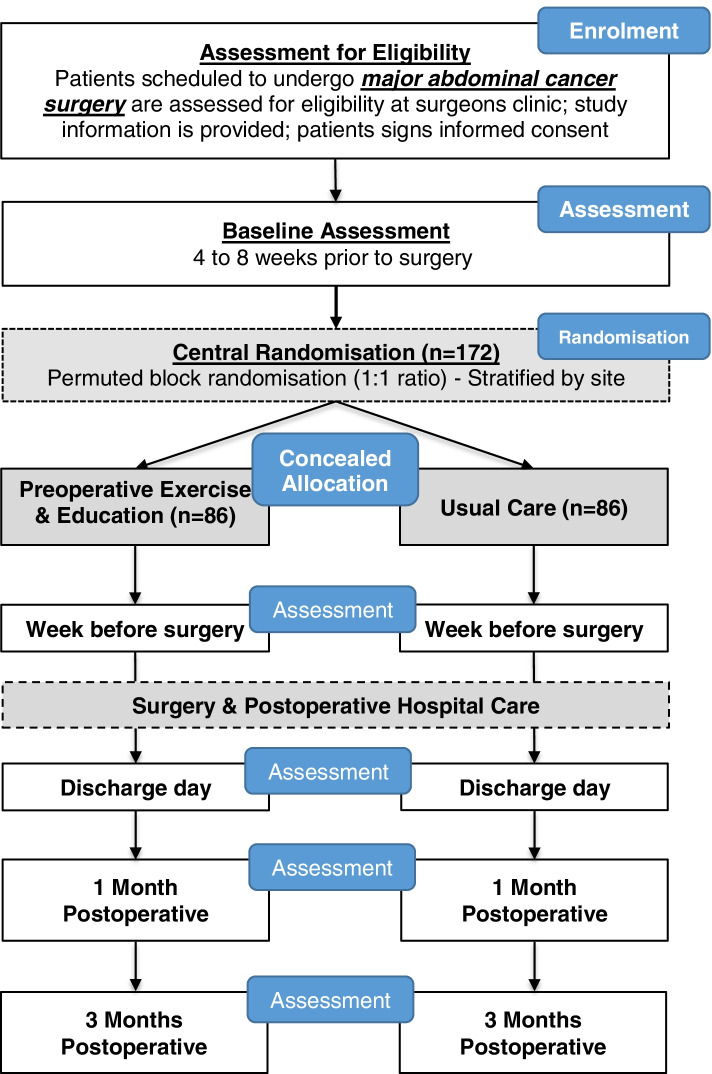
PRIORITY TRIAL flow chart

The proposed study is a multi-centre, assessor-blinded, pragmatic, comparative, RCT, with two parallel groups randomised in a 1:1 allocation. Participants will be randomised to (i) a progressive, preoperative exercise and education program delivered over 4 to 8 weeks prior to surgery or (ii) usual perioperative care alone (Fig. 1).

### Study sites

This multicentre trial will be performed in three centres in Australia: Royal Prince Alfred Hospital (leading trial site) and the Chris O’Brien Lifehouse (satellite site) in Sydney, and the Peter MacCallum Cancer Centre in Melbourne. These sites are nationally recognised as leading centres for managing patients undergoing major advanced primary or recurrent gastrointestinal cancer surgery in Australia.

### Participants

A total of 172 consecutive participants undergoing major advanced primary or recurrent gastrointestinal cancer surgery will be included. To be eligible to enter this trial, all participants must satisfy the following inclusion and exclusion criteria:

Inclusion criteria:

(i) Adult patients (18-80 years) undergoing elective major surgery for advanced or recurrent gastrointestinal cancer, including pelvic exenteration (advanced primary or recurrent pelvic cancers), cytoreductive surgery, oesophagectomy, hepatectomy, gastrectomy or pancreatectomy;
(ii) Consult a gastrointestinal surgeon at least 4 weeks before planned surgery.

Exclusion criteria:

(i) Cognitive impairment such that they are unable to provide informed consent;
(ii) Co-morbidity preventing participation in exercise and physical assessments (i.e., cardiorespiratory, musculoskeletal, neurological);
(iii) Presenting with very poor functional capacity. Defined as a score of ‘zero’ (i.e., No) to all the following Duke Activity Status Index (DASI)(31) questions:
  - Can you climb a flight of stairs or walk up a hill?;
  - Can you do heavy work around the house?;
  - Can you do yard work?;
  - Can you participate in strenuous sports?

(iv) Inadequate English language to complete outcome measures;
(v) Currently participating in an exercise program.

### Participant recruitment and mode of contact

Surgeons, Anaesthetists, Allied Health practitioners, Clinical Research Managers, and Research Officers from the participating institutions will be involved in patient identification and screening. All patients will be screened for inclusion and exclusion criteria. Eligible patients will be provided with a patient information sheet. If they are interested in participating, they will provide consent for a researcher from the trial to contact them and discuss the trial further. An adequate time (24 h from initial contact) will be provided for all patients to understand and consider what the trial is proposing and for their questions and concerns to be addressed. After this period, the trial’s research officer will seek consent from patients. Clinical assessment and decision-making will proceed according to usual clinical protocols. The number of patients screened and reasons for ineligibility in the study will be documented. After signed consent is obtained, participants will be contacted by an authorised member of the study team at baseline (preoperatively), during the intervention period (4-8 weeks before the surgery), in-hospital, and up to 3 months post-operatively, either via face-to-face contact, telephone, text message, e-mail or post, accordingly to the patients’ preferences. During this time, demographic, clinical, and data referent to the trials’ assessment schedules will be collected. Participation in the PRIORITY Trial will not affect surgical waiting time. Recruited participants may choose to withdraw from the study at any stage. All data collected before the time of study withdrawal will be included in data analyses, including clinical information routinely collected during their in-hospital care (e.g., postoperative complications and length of stay).

### Randomisation and blinding

Consenting participants will undergo baseline assessment immediately before randomisation to intervention (prehabilitation with a progressive preoperative exercise and education program) or control group (usual care alone). An experienced biostatistician, not involved in patient recruitment or data collection, will create the randomisation sequence a priori using a central secure (computerised) randomisation service (1:1 allocation ratio in random permuted blocks of sizes 4 or 2 stratified by site, to ensure balance in treatment assignment within the study sites) to ensure concealment of treatment allocation and blinding of the treating surgeon, all trial personnel and the statistician. The randomisation schedule (i.e., allocation tables as Microsoft Excel file) will be uploaded by an independent Research Officer (not involved in the trial) into the PRIORITY TRIAL’s Research Electronic Data Capture (REDCap) database. The REDCap randomisation module will randomise participants into the intervention or control group. Due to the nature of the intervention, it is not possible to blind the clinicians delivering the intervention (exercise and education program); however, we will ensure that all patients’ interactions are standardised.

### Interventions

Participants will be allocated to prehabilitation with an individualised progressive preoperative exercise and education program (intervention group) or usual care alone (control group).

#### Intervention group: Prehabilitation with individualised preoperative exercise and education program

Registered clinicians will deliver the intervention program (e.g., physiotherapists or exercise physiologists) in existing community-based clinics close to the study participant’s home. A video and a written manual will be provided to the physiotherapists and exercise physiologists delivering the exercise and education program to ensure standardisation of treatment. The video and written manual will cover the background information on preoperative exercise, trial’s aims, methodology, including information on the surgical procedures, the preoperative exercise, education program, safety, and required trial documentation and paperwork. The main aim of the exercise and education program is to increase aerobic capacity and skeletal muscle endurance, muscle strength, and respiratory muscle function and to educate participants on how to perform a component of the exercise program at home and about the surgical process. Patients will complete supervised exercise training sessions with registered clinicians (3 days per week), home-based functional exercises prescribed by the treating clinician (4 days per week), and daily physical activity to be encouraged to walk for at least 30 min per weekday. Patients will be provided an activity monitor for the program duration.

The individualised exercise program will follow the FITT-VP (frequency, intensity, time, type, volume, and progression) principles suggested by the American College of Sports Medicine [31] and will be delivered using three main components, including (i) supervised, individualised, progressive exercise program, (ii) home exercises, and (iii) daily physical activity advice as detailed in the Template for Intervention Description and Replication (TIDieR) [32] checklist (Table 1). The PRIORITY Trial will cover all the expenses for patients that are related to the training sessions. The adherence to the exercise program will be encouraged by the treating physiotherapist or exercise physiologist and by a research team member that will message or call patients to check their compliance. The team member’s method of contact will depend on patient preferences. Treating clinicians and the study participants will be provided with an activity diary. They will be encouraged by the trials’ research officer through weekly reminders to complete the activity diary daily.

**Table 1:** Description of the intervention using the Template for Intervention Description and Replication (TIDieR) Checklist

Brief name	PRIORITY TRIAL
**Why**	Radical surgery is the only treatment option that offers a chance of survival for patients presenting with major advanced or recurrent gastrointestinal cancer. While these procedures significantly improve survival, rates of major postoperative complications remain high. An exercise program to improve physical conditioning before surgery could enhance recovery and reduce postoperative complications among patients undergoing surgery. These patients routinely have a period of 4-8 weeks from first surgical consultation to undergoing the procedure, which provides an opportunity to deliver a tailored, progressive exercise and education program.
**What materials?**	Participants allocated to the intervention group will receive an individualised exercise and education program during the preoperative period delivered by community-based physiotherapists and exercise physiologists. It’s expected that the physiotherapists and exercise physiologists will use various materials, such as weights, elastic bands, stationary bicycles, treadmills, to deliver the proposed intervention. Further, participants will be encouraged to walk continuously for at least 30 min daily, using an activity monitor (Yamax Power Walker EX-510). Participants from the control group will be given an activity monitor for 1 week before surgery only. This is to measure their objective level of physical activity during the preoperative period. The screen will be blocked with security tape, and all feedback and incentivising features will be deactivated.
**What procedures?**	**INDIVIDUALISED PREOPERATIVE EXERCISE AND EDUCATION PROGRAM (Intervention)** The intervention will be delivered using three main components: **1) Supervised, individualised, progressive exercise program**: The exercise program will consist of a 50 min individualised (one-to-one) training session with a physiotherapist or exercise physiologist, three times a week, for 4 to 8 weeks (range from 12 to 24 sessions). Each session will include warm-up, aerobic/anaerobic, resistance, respiratory exercises, and cooldown activities: ***a. Warm-up*** (~ 5 min): Low-intensity exercises (e.g., walking, cycling) ***b. Exercises*** (~ 40 min): *i. Aerobic and anaerobic exercises (~ 15-25 min)*: Cycle ergometer, treadmill, or rowing machine (high-intensity interval training based on their rated perceived exertion – M-Borg scale). This will be adjusted to the response of the patient as they progress through the exercise sessions. Considerable flexibility with the prescription of the high-intensity interval training will be adopted to allow for individual participant responses. Phase 1 (approximately 2 weeks): Exercise intensity focused on rated perceived exertion of 3 to 5 on the M-Borg Scale continuous 20 min session or 2 × 10 min sessions (Low intensity). This phase is ideal to prepare the patient for Phase 2. Sedentary patient may need to stay in this phase for approximately 2 weeks, while more active patients can move to Phase 2 sooner. Phase 2 (approximately 2-4 weeks): The intensity and duration of the exercise will be increased gradually. Exercises will be focused on a rated perceived exertion of 3 to 5 on the M-Borg Scale for 30-40 min (Moderate intensity). Once the patients can tolerate the Phase 2 sessions at the abovementioned M-Borg scores, they could progress to interval training at a higher intensity (Phase 3). Phase 3 (approximately 2-4 weeks): Introduce anaerobic high-intensity interval training at 5 to 8 on the M-Borg Scale (4 high intensity intervals on the M-Borg Scale of 5-8 lasting 4 min each with interval reduction of intensity on the M-Borg Scale of 3-5 for 4 min). *ii. Respiratory exercises* (~ 5-10 min): Deep breathing exercises alone or in a cycle with relaxed breathing, huff, and cough (Active Cycle of Breathing Technique). *iii. Resistance exercises (~ 10-20 min):* Squat, push-up, shoulder press, hamstrings curl, dumbbell deadlift, biceps curl, and overhead triceps extension (performed at 40-60% of the one-repetition maximum). ***c. Cooldown*** (~ 5 min): Stretching and flexibility exercises (Triceps, lower back, hip flexors, quadriceps, hamstrings, calf muscles, light jogging, walking, or stretching exercises), performed twice for 20-30 s. **2) Home exercises (4 times/week):** Home-based functional exercises will be prescribed by the treating physiotherapist or exercise physiologist to increase aerobic capacity and respiratory muscle function, including exercises with resistance provided by bodyweight, including push-ups, squats, step-ups. On the day’s participants are not attending the exercise training session, they will be instructed to perform the exercises for 30 min at home. **3) Daily physical activity advice**: In addition to home exercises, participants will be encouraged to walk continuously for at least 30 min daily, using an activity monitor (Yamax Power Walker EX-510). Participants will be asked to keep a daily diary of the number of steps per day and the number of home exercises performed during the preoperative period. The activity monitor will be provided by the study research team free of charge after the baseline assessment. The device will be collected from the patient at the following assessment (the week before surgery). Step count will be extracted from the activity monitor daily by the participant, and the research team will check all activity monitor data once the device has been returned. Access to this information will be limited to the patient and the research team. Once participation has been completed, the research team will delete all data from the activity monitor. The information stored on the devices will be erased. No one apart from the study investigators will have access to the data collected. No data collected from the activity monitor will be sent to any of the involved institutions. The results from the study will not be used to register the activity monitor. **USUAL CARE (CONTROL GROUP)** Participants allocated to the control group will receive usual care consisted of nutritional counselling and advice on smoking cessation and reduction of alcohol intake. However, no exercise advice will be provided, and patients will be instructed to maintain their normal daily activities.
**Who provided?**	The intervention will be administered by local registered physiotherapists or exercise physiologists who will educate and provide high-intensity interval training, home exercises prescription and progression, and physical activity advice per the study protocol.
**How?**	The exercise program (supervised, individualised, progressive exercise program) will be individualised and delivered by registered physiotherapists or exercise physiologists. The home exercises will be prescribed by the registered physiotherapists or exercise physiologists and will be conducted at home, unsupervised. Likewise, the registered physiotherapists or exercise physiologists will give the daily physical activity advice to be completed by the participant, unsupervised.
**Where?**	The intervention will be conducted at private clinics, mainly in Sydney (New South Wales) and Melbourne (Victoria), Australia. However, as the trial is recruiting from major referral hospitals, it is expected that a small proportion of the patients will reside outside of New South Wales and Victoria. Therefore the intervention may also be conducted in private clinics in other Australian states.
**When and how much?**	The preoperative exercise and education program will be delivered 4-8 weeks before surgery and include: (i) Up to 24 sessions × 50 min: Supervised, high-intensity training. Individualised exercise prescription, progression, and follow-up with a local physiotherapist or exercise physiologist; (ii) Up to 32 sessions × 30 min: Unsupervised home exercise; (iii) Up to 56 walking sessions × 30 min: Advice to walk continuously (30 min daily).
**Tailoring**	The exercise program will be tailored to each participant based on a health assessment, considering patients’ current health status, physical activity level, presence of co-morbidities, and medical history.

#### Control group: usual perioperative care

Participants allocated to the control group will receive usual care consisting of nutritional counselling and advice on smoking cessation and reduction of alcohol intake where relevant. No exercise advice will be provided, and patients will be instructed to maintain their normal daily activities.

To measure their objective level of physical activity during the preoperative period, study participants will be sent an activity monitor to wear in the week before their second assessment. Participants will be asked to maintain their usual activities during this period. All feedback and incentivising features on the monitor be deactivated.

### Treatment fidelity

To ensure the intervention is delivered according to protocol, we will create manuals of procedures and provide training to all staff involved in providing the study treatment and assessment of participants. To ensure standardisation, we will hold weekly meetings across the study sites. An independent researcher will audit a randomly chosen subset of patients to ensure adherence to the intervention program and the study protocol. This method was successful in our pilot study (92.7%) [33].

### Outcome measures

The following primary, secondary and other outcome measures will be collected across five time points: during the preoperative period (baseline assessment), within a week prior to the scheduled surgery (post-intervention), and postoperatively on the day of discharge from hospital, 1 month and 3 months after surgery (Table 2).

**Table 2:**
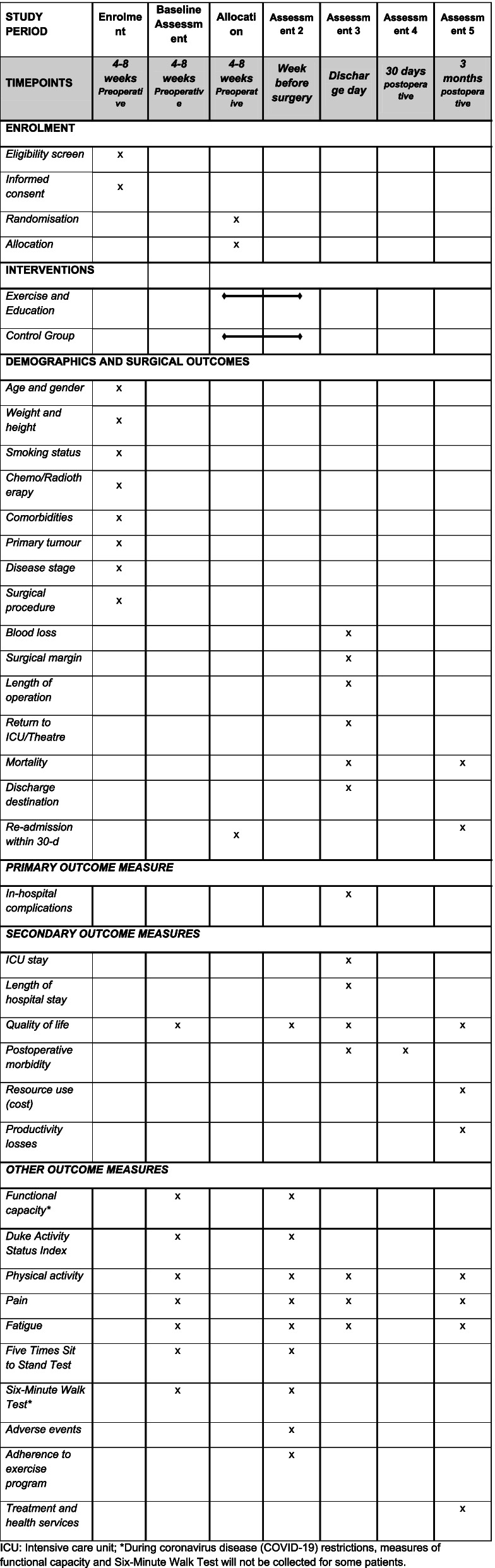
Details of the schedule of enrolment, interventions and assessments according to Standard Protocol Items: Recommendations for Intervention Trials (SPIRIT) Diagram

#### Primary outcome measure

The primary outcome will be the proportion of participants developing ***in-hospital complications*** within the primary admission of the index surgery. The definition of each postoperative complication will be described per the Standardised Endpoints Core Outcome Measures in Perioperative and Anaesthetic Care (StEP-COMPAQ) group [34]. The primary outcome will be independently extracted from the hospital electronic medical records by two trial personnel at each study site blinded to group allocation. Disagreements will be resolved by discussion.

#### Secondary outcome measures

The secondary outcomes will be:

(i) *Intensive care unit and hospital length of stay:* The duration of stay in the intensive care unit and hospital (in days); with the day of the index surgery considered as day 0. This information will be extracted from the hospital’s electronic medical records.
(ii) *Quality of life*: measured by the Short Form 36 version 2 (SF-36v2®, Australian English) [35] at baseline (4-8 weeks preoperative), within the week before surgery, discharge day, and 3 months postoperative. The QualityMetric Health Outcomes™ Scoring Software will be used to obtain the final scores of the eight quality of life domains (physical functioning, role physical, bodily pain, general health, vitality, social functioning, role emotional, and mental health) and the two physical and mental summary scores.
(iii) *Postoperative morbidity*: Further investigations on postoperative morbidity will be conducted as a secondary outcome measure:
  - *Morbidity on postoperative day five and postoperative day 30*: Morbidity on postoperative day 5 (or the day of discharge if the participant is discharged earlier) will be assessed by the Postoperative Morbidity Survey (POMS) [36] and morbidity on postoperative day 30 will be captured using the postoperative complication definition recommended by the StEP-COMPAQ group [34]. The severity of each complication will be graded using Clavien-Dindo classification [37, 38]. The overall postoperative morbidity will also be expressed using the Comprehensive Complication Index (CCI) [39] which weights patients with multiple complications higher than patients with only a single complication yet of the same highest Clavien-Dindo severity grade.
  - *Days at home within first 30 days after surgery (DAH-30)*: DAH-30 is a composite measure that accounts for the length of stay in the hospital following index surgery (Day 0), readmission to either the index or any other hospital, hospital discharge to a rehabilitation centre/hospital or nursing facility, and early deaths after surgery within a single metric. DAH-30 (number, value between 0 and 30) will be calculated using mortality and hospitalisation data from the date of the index surgery (day 0). For example, if a patient dies on postoperative day three, they will be assigned 0 for DAH-30. If a patient is discharged from the hospital on day six after surgery but is subsequently readmitted for 4 days before their second hospital discharge, the patient will be assigned 20 (=30-6-4) DAH-30. If a patient has complications and spends 16 days in the hospital, then is transferred to a nursing facility for rehabilitation, and spends 24 days there before finally being discharged to their own home, the patient would be assigned 0 (30-16-24 = − 10, set to 0) for DAH-30. If a patient dies within 30 days of surgery, irrespective of whether the patient has spent some time at home, DAH-30 will be scored as zero (0) [40, 41].
(iv) *Health resource use (costs) and productivity losses*: Information on patient out-of-pocket and health system resource use (in Australian dollars, AUD), including the cost of the exercise intervention, will be collected (via follow-up questionnaires) from randomisation up to 3 months postoperative [11]. Information about the impact of the intervention and surgery on return to work and leisure activities will be used to inform productivity losses.

#### Other outcome measures

The following outcome measures will be collected:

(i) *Functional capacity*: This will be measured by the CPET at baseline and within 1 week before the scheduled surgery [42]. Improved fitness will be defined as a positive change (≥10% on peak VO_2_, to account for inter-test variability) between CPET tests. Due to coronavirus disease (COVID-19) outbreaks in Australia, some clinics may not be able to see participants face-to-face, and access to CPET will be restricted and will not be uniformly available to all participants.
(ii) Duke Activity Status Index (DASI): functional capacity will be measured at baseline and within a week before surgery. The DASI is a 12-item scale in the form of a self-reported questionnaire. The final score ranges from 0 to 58, where a higher score indicates higher functional capacity [43].
(iii) *Self-reported physical activity*: Self-reported physical activity will be measured at baseline, within a week before surgery, and postoperatively on discharge day and after 3 months using the following tools: The Active Australia Survey [44], strength questions from the New South Wales Health population survey (https://www.health.nsw.gov.au/surveys/adult/Pages/default.aspx) and sitting questions from the International Physical Activity Questionnaire short form (IPAQ-SF) [45].
(iv) *Objective physical activity*: Objective physical activity will be measured during the preoperative period using an activity monitor (Yamax EX510 Activity Tracker). For the intervention group, the activity monitor will be used during the whole intervention period and will provide real-time visual feedback. For the control group, objective physical activity will be collected using an activity monitor without any visual feedback during the 7 days before their second assessment (within 1 week before the scheduled surgery).
(v) *Pain intensity*: Pain will be measured on a numerical pain rating scale (NPRS; 0 to 10 - where higher scores indicate worst symptoms) [46] at baseline, within a week before surgery, and postoperatively on discharge day and after 3 months.
(vi) *Satisfaction*: Patient satisfaction of the intervention will be measured at the follow-up visit within 1 week before surgery using a self-reported questionnaire [33].
(vii) *Fatigue*: Fatigue will be measured using the Fatigue Severity Scale at baseline, within 1 week before surgery and postoperatively on discharge day and after 3 months [47].
(viii) *Five Times Sit-to-Stand Test*: Lower limb strength and function will be measured using the five times sit-to-stand test [48]. Patients will sit with arms folded across their chest and back against a chair. Then, patients will be instructed to stand up and sit down five times as quickly as possible, and the time taken to complete the test will be recorded. Due to COVID-19 outbreaks in Australia, participants will have the option to perform this assessment via video call.
(ix) *Six-Minute Walk Test (6-MWT)*: Aerobic capacity and endurance will be measured using the 6-MWT. The test will be performed using the American Thoracic Society (ATS) guidelines indoors, along a flat, straight, enclosed corridor at each participating site [49]. The course will be 30 m in length and marked every three meters. The starting line and turning points will be marked and indicated to patients. Patients will be instructed to rest before beginning the test near the starting position, a forehead sensor and pulse oximeter will be attached to the patient for SpO2 and heart rate measurements, a timer will be set to 6 min, and a standardised guide will be used to provide instructions to patients. Patients will be informed that the objective of the test is to walk as far as possible for 6 minutes but are permitted to slow down, stop or rest when required. The distance walked will be recorded. Due to COVID-19 outbreaks in Australia, some clinics may not be able to see participants face-to-face. Thus, the 6-MWT assessment will be restricted and will not be uniformly available to all participants.
(x) *Adherence to the study exercise program*: Defined as the percentage of exercise sessions attended/performed by those patients randomised to the preoperative intervention group. This will be collected using the clinician activity record. In addition, participants will receive the participant exercise diary to document their supervised sessions, home exercises, and physical activity advice. Participants will be contacted every week by the trials’ research officer to report their adherence to the preoperative program. Successful adherence to the preoperative program will be defined as ≥70% attendance of the supervised sessions.
(xi) *Adverse events*: Defined as any undesirable event, such as injury, fall, severe breathlessness, new or progressive pain, and progressive fatigue will be collected preoperatively during or up to 60 min following the trial intervention or outcome assessment. Serious adverse events will be defined as life-threatening or result in disability, incapacity, hospitalisation, or death during or up to 60 min following the trial intervention or outcome assessment. In serious adverse events, the trial chief investigator and trial research officer will be notified immediately, and the incident reported to the relevant hospital ethics committee.
(xii) *Treatment and Health Services*: Use of treatment and health services will be collected (via follow-up questionnaire) up to 3 months after surgery for the cost-effectiveness analysis. This would include information about treatments (physiotherapy, etc.), diagnostic imaging, visiting GP/specialist clinics, emergency department visits, and hospital readmissions.

### Reporting of missing data

The Patient-Reported Outcome Completion and Missing Data (PRO CoMiDa) [50] is a data management tool designed to provide standardised documentation of the completion or reasons for non-completion of patient-reported outcomes assessments by patients in a clinical trial. The form will be completed at each scheduled patient-reported outcomes assessment.

### Data management

All data will be collected and managed using a REDCap [51, 52] database by a blinded research team member. REDCap is a secure web-based application for building and managing online databases and is licensed and supported by the Sydney Local Health District. A digital copy of completed questionnaire forms will also be scanned and uploaded by the study research team onto the Royal Prince Alfred Hospital, Sydney Local Health District secure server. An independent research officer will double-check uploaded records. Hard copy questionnaire forms will be shredded after they have been uploaded. To minimise potential bias arising from missing data at random, multiple imputations will be performed.

### Sample size calculation

To achieve the study’s primary aim, 172 participants will provide 90% power to detect a difference of 25% in complication rates between the intervention and control groups. This difference was considered clinically meaningful by our clinical and consumer panel. These calculations were based upon the overall complication rate being 50% in the control groups, based on our current clinical experience, allowing for up to 5% loss to follow-up and a two-sided alpha of 0.05. The 5% loss to follow-up was estimated from our previous prospective clinical trials [26]. Sample size calculations were conducted using PASS15 software (NCSS LLC).

### Analyses

A comprehensive Statistical Analysis Plan (SAP) and a Health Economic Analysis Plan (HEAP) will be developed by the trial statistician and health economists before database unlocking.

Data analysis will be blinded, following the intention-to-treat principles and guided by a detailed statistical analysis plan. We will restrict the number of analyses to reduce the possibility of Type I errors. Analysis and interpretation (also performed blinded) on the primary and secondary outcomes will be conducted by two independent biostatisticians and checked for accuracy. Demographic and clinical characteristics of the patients in each group will be compared to assess the comparability of the groups. For the primary outcome, a *p*-value of < 0.05 will be considered statistically significant. For the secondary outcomes, a p-value of < 0.01 will be considered significant.

*Primary analysis* (comparison of in-hospital complication rate between groups): Proportions and 95% confidence intervals for in-hospital postoperative complications will be calculated. Differences in this outcome will be compared between intervention and control groups using the chi-square test. *Secondary analysis* (comparison of hospital stay postoperative morbidity and quality of life between groups): Mean and standard deviation (or median [IQR] if applicable) for intensive care and hospital length of stay, postoperative morbidity (including POMS at 5 days postoperative), DAH-30 and quality of life will be calculated. The difference in these outcomes will be compared between the intervention and control groups using an independent sample t-test for normal distributed data and Mann-Whitney U-test for non-normally distributed data. Per-protocol post hoc analyses will be performed to further explore the effects of preoperative exercise and education on the primary and secondary outcomes compared to a control group. Adherence to the preoperative exercise program will be defined as ≥70% attendance of the supervised sessions. To improve the precision of the estimates and to compensate for potential imbalances, estimates will be adjusted for age, sex, type of cancer, preoperative chemo/radiotherapy, and other baseline parameters. Both adjusted and unadjusted estimates will be reported. Linear regression and logistic regression will be used to compare outcomes between groups.

### Economic evaluation

The economic evaluation will consist of within-trial cost-effectiveness and cost-utility analyses. Costs will be measured in terms of direct costs to the healthcare system and out-of-pocket costs to patients over the three-month study period, using previously developed case report forms and monthly patient diaries [53]. Outcomes will include postoperative complications (primary outcome) and quality-adjusted life-years (QALYs). Utilities for quality of life adjustment will be taken from the SF-36 questionnaire containing the SF-12 instrument and converted to SF-6D scores [54]. Both costs and outcomes will be reported separately for each group (following best practice methods) [55] and an incremental cost-effectiveness ratio (ICER) will be calculated for (i) the additional cost of a pre-operative exercise program per in-hospital complication avoided; and (ii) the additional cost per QALY gained at 3 months. Presentation of results in this way will facilitate reimbursement decision-making for pre-operative physiotherapy/exercise physiology services. In addition, a separate estimate on the financial burden of surgical treatment from a patient’s perspective will be reported.

### Data and safety monitoring

An independent Data and Safety Monitoring Board (DSMB), led by an experienced physiotherapist, a gastrointestinal surgeon, and a statistician, will monitor adverse events and adherence to the protocol at regular intervals to ensure the safety of participants. The frequency of DSMB meetings and the stopping rules for the study will be defined a priori in a charter, in consultation with the DSMB members and study investigators.

### Dissemination of results

The results of this trial will be submitted for publication in international or national peer-reviewed journals with all collaborators acknowledged. Results will also be disseminated through conference presentations and general media. It is expected that several publications will originate from this protocol, addressing the aims as mentioned above. All publications arising from this protocol will be reported on the Ethics annual report. Anonymised data will be made available upon reasonable request and in accordance with the Sydney Local Health District Human Research Ethics Committee.

### Trial status

The PRIORITY Trial started recruitment in June 2021. Due to COVID-19 restrictions, recruitment was paused in September 2021 (No participant was recruited within this period). A COVID-19 contingency plan was developed and participant recruitment was resumed in November 2021. The completion of this trial is expected by the end of 2024.

## Discussion

Advances in surgical skills, devices, medical treatment, and intensive care have led to dramatic improvements in the 5-year survival for people who undergo major advanced or recurrent gastrointestinal cancer surgery. However, as the surgical complexity and the patient demographic of increasing age and burden of comorbid disease increases, we face an ongoing challenge of a high incidence of postoperative complication rates. Reducing this high incidence of postoperative complication rates represents the next challenge to improve surgical outcomes for patients.

The PRIORITY Trial will rigorously assess whether a progressive, individualised prehabilitation with preoperative exercise and education as an intervention to improve patients’ fitness can significantly reduce postoperative complication rates. There are direct benefits if a positive result is achieved in the PRIORITY Trial. For instance, a patient with one postoperative complication spends an extra 7 days in the hospital, and for those with ≥2 complications, the associated costs are increased by seven-fold. Therefore, a reduction in postoperative complication rates will likely reduce the average length of hospital stay and consequently improve postoperative quality of life. If successful, this intervention will improve patient outcomes and may reduce healthcare expenditure. Furthermore, the intervention could be adapted for patients undergoing other major procedures for malignant or non-malignant disease.

Some of the main strengths of the PRIORITY Trial include the delivery of a structured preoperative exercise program by registered, community-based physiotherapists and exercise physiologists. This not only broadens the inclusion criteria to include patients who live far away from major specialised hospitals (including rural/remote areas), but it also tests the generalisability and scalability of the intervention, as it is delivered in the local community setting and at-home. A survey of patients’ perspectives confirmed the desire to undertake exercise and prehabilitation outside of the hospital setting [56]. Due to the nature of the intervention, a limitation of this trial is that participants and the physiotherapist and exercises physiologists delivering the intervention will not be blinded.

In summary, the PRIORITY Trial will provide important information to guide preoperative optimisation strategies and has the potential to reduce postoperative complication rates and length of hospital stay, having the potential to save millions of dollars that can be redirected to other areas of health and to improve the life of patients undergoing radical surgical procedures significantly.

## Data Availability

At completion, data originated from the PRIORITY Trial will not be publicly available accordingly to the legislation of the Sydney Local Health District Human Research Ethics Committee, but anonymised data are available from the corresponding author upon reasonable request.

## Abbreviations

CONSORT: Consolidated Standard of Reporting Trials
SPIRIT: Standard Protocol Items: Recommendations for Interventional Trials
Peak VO_2_: Peak oxygen consumption
AT: Anaerobic threshold
CPET: Cardiorespiratory Exercise Test
COVID-19: Coronavirus Disease
REDCap: Research Electronic Data Capture
FITT-VP: Frequency, intensity, time, type, volume and progression
StEP-COMPAQ: Standardised Endpoints Core Outcome Measures in Perioperative and Anaesthetic Care
TIDieR: Template for Intervention Description and Replication
SF-36v2: Short Form 36 version 2
POMS: Postoperative Morbidity Survey
CCI: Comprehensive Complication Index
DAH-30: Days at home within first 30 days after surgery
AUD: Australian dollars
IPAQ-SF: International Physical Activity Questionnaire short form
NPRS: Numerical pain rating scale
ATS: American Thoracic Society
PRO CoMiDa: Patient-Reported Outcome Completion and Missing Data
QALYs: Quality adjusted life years
ICER: Incremental cost-effectiveness ratio
SAP: Statistical Analysis Plan
HEAP: Health Economic Analysis Plan
DSMB: Data and Safety Monitoring Board
